# AlloPred: prediction of allosteric pockets on proteins using normal mode perturbation analysis

**DOI:** 10.1186/s12859-015-0771-1

**Published:** 2015-10-23

**Authors:** Joe G Greener, Michael JE Sternberg

**Affiliations:** Centre for Integrative Systems Biology and Bioinformatics, Department of Life Sciences, Imperial College London, London, SW7 2AZ UK

**Keywords:** Allostery, Normal modes, Pocket prediction, Machine learning, Web server

## Abstract

**Background:**

Despite being hugely important in biological processes, allostery is poorly understood and no universal mechanism has been discovered. Allosteric drugs are a largely unexplored prospect with many potential advantages over orthosteric drugs. Computational methods to predict allosteric sites on proteins are needed to aid the discovery of allosteric drugs, as well as to advance our fundamental understanding of allostery.

**Results:**

AlloPred, a novel method to predict allosteric pockets on proteins, was developed. AlloPred uses perturbation of normal modes alongside pocket descriptors in a machine learning approach that ranks the pockets on a protein. AlloPred ranked an allosteric pocket top for 23 out of 40 known allosteric proteins, showing comparable and complementary performance to two existing methods. In 28 of 40 cases an allosteric pocket was ranked first or second. The AlloPred web server, freely available at http://www.sbg.bio.ic.ac.uk/allopred/home, allows visualisation and analysis of predictions. The source code and dataset information are also available from this site.

**Conclusions:**

Perturbation of normal modes can enhance our ability to predict allosteric sites on proteins. Computational methods such as AlloPred assist drug discovery efforts by suggesting sites on proteins for further experimental study.

## Background

Allostery is a process where one site on a molecule is perturbed by an effector, causing a functional change at another site: it is regulation at a distance [1]. Allostery can arise from non-covalent interactions (e.g. drug binding), covalent interactions (e.g. phosphorylation) and light absorption. This intrinsic and widespread property of proteins [2] is important in processes such as cellular signalling and disease, yet most allosteric mechanisms remain an enigma and a universal mechanism has proved elusive [3, 4].

Allosteric drugs have hardly been explored and are a major avenue of research for the pharmaceutical industry [5–7]. They hold many potential benefits over orthosteric (non-allosteric) drugs: they do not bind to active sites that are often conserved in protein families, and are hence highly specific; they can activate as well as inhibit a protein; they can have a ceiling to their effect; and they can be used effectively in combination with orthosteric drugs. However, discovery of allosteric drugs presents challenges beyond those encountered in orthosteric drug discovery. Whether the drug will activate or inhibit the protein is difficult to predict and in many cases the location of allosteric sites is unknown.

Allosteric drug discovery by virtual screening is an exciting prospect furthered by the elucidation of previously-unknown allosteric sites found on solved protein structures [8]. Development of allosteric prediction methods is therefore of pressing concern and has been approached using various methods: changes in flexibility on ligand binding [9, 10]; machine-learning using pocket features [11]; structural conservation [8]; two-state Gō models [12]; and molecular dynamics [13]. Methods investigating the allosteric mechanism have also been developed [14–17], giving insight into which residues propagate the allosteric signal and how it is transmitted. Many of the above approaches have been made available to the community as web servers [11, 18–20].

Several studies have used normal mode analysis (NMA) to model allosteric regulation [9, 10, 16, 21, 22]. In NMA, the structural fluctuations of a protein around an equilibrium conformation are decomposed into harmonic orthogonal modes [23]. NMA is effective at describing protein dynamics, despite ignoring the complex nature of the protein energy landscape [24]. Even considering the C-alpha atoms alone can be sufficient. The long-range nature of allosteric communication is often well-described by low-frequency modes that involve the motion of many atoms, though allostery does involve local effects so higher-frequency modes should also be taken into account [25].

We developed a novel procedure, AlloPred, which uses NMA to predict the allosteric pockets on a protein. AlloPred models how the dynamics of a protein would be altered in the presence of a modulator at a specific pocket. Pockets on the protein were first predicted using the Fpocket algorithm [26], which locates pockets using Voronoi tessellation and alpha spheres. The normal modes of the protein were then calculated using the elastic network model, except the spring constant of any atom pair including a residue in a chosen pocket was set to be a higher value. The effect of this perturbation was measured at the active site. These results were combined with output from Fpocket in a support vector machine (SVM) to predict allosteric pockets on proteins.

## Methods

### Data selection

ASBench [27], a benchmarking set for allosteric discovery, was used as a source of known allosteric proteins. The ‘Core-Diversity set’ contains 147 structurally-diverse allosteric sites on 127 proteins from a variety of protein classes such as transferases, hydrolases and transcription factors. The PDB files, allosteric site data and active site data were obtained for each protein from ASBench. UniProt [28] and the Catalytic Site Atlas [29] were used to find active site data when it was not available from ASBench. In each PDB file, only the chain(s) containing the active and allosteric sites, and any chains linking them, were considered. This was in order to keep the size of the proteins manageable, as using entire protein assemblies would lead to a large number of pockets. It also allowed comparison with existing methods, which use similar criteria. In practice the use of larger assemblies was tried during development and did not have a large effect on the results. Seven proteins were removed from the set as the PDB file did not contain the active site, i.e. the PDB file represented the allosteric section of a larger protein. One protein was removed as Fpocket did not run successfully. This left 119 proteins in the dataset. The dataset was randomly split into a training set of 79 proteins and a test set of 40 proteins.

### Pocket prediction

Potential binding pockets on the proteins were calculated using the open-source Fpocket v2.0 algorithm, which has been shown to be effective in comparison to other methods [26]. The default parameters used in the Fpocket calculation produced pockets that were large enough to place most (average 86 %) allosteric binding residues in pockets but not so large that identifying a pocket as having allosteric effect was of little use. Sometimes multiple allosteric pockets on the same protein represented different and physically-separated allosteric sites, and sometimes adjacent calculated pockets covered a single allosteric binding site. The pockets also covered much of the protein surface, which allowed the method to detect allosteric sites that could be found anywhere on the surface. On average 41 % of residues in each protein appeared in a pocket.

Fpocket output 2,201 pockets for the 119 proteins (average 18.5 per protein), of which 389 (18 % of pockets, average 3.3 per protein) contained at least one residue identified as binding to an allosteric modulator and were hence labelled as *allosteric pockets*. Although being defined as an allosteric pocket in this manner does not necessarily mean that binding to that pocket causes the allosteric effect, the average number of allosteric binding residues in an allosteric pocket was 4.3, indicating the utility of locating such pockets. All but 5 proteins in the dataset had at least one allosteric binding residue placed in a pocket. We treated pockets without known allosteric binding residues as negative examples during machine learning. It should be noted that these pockets may not correspond directly to the actual pockets on the protein, or may have latent allosteric character yet to be discovered.

### Normal mode analysis

In NMA the Hessian matrix - the matrix of second derivatives of the potential energy *V* with respect to the mass-weighted atomic coordinates - is diagonalised to yield the normal modes [23]. The potential energy *V* was described according to the elastic network model [30] as a set of harmonic springs of strength *k* between every pair of C-alpha atoms no further than distance *R*_*c*_ apart:

$$V = \sum_{\substack{r_{ij}^{0} < R_{c} \\ i < j}} k \left(r_{ij} - r_{ij}^{0}\right)^{2} $$

where $r_{\textit {ij}}^{0}$ is the Euclidean distance between atoms *i* and *j* in the PDB file. We used values of 1 kcal mol^-1^ Å^-2^ and 15 Å for *k* and *R*_*c*_ respectively.

**Fig. 1:**
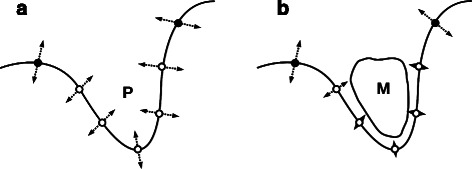
Change in flexibility on modulator binding. Diagram showing the change in flexibility of a protein on modulator binding at an allosteric site. The solid line indicates the surface of the protein and circles show residues: non-filled circles represent residues that are part of pocket P and filled circles represent other residues. Dashed arrows represent the magnitude of the fluctuations of a residue about equilibrium. **a** shows the protein in the absence of a modulator. All residues can vibrate. **b** shows the effect of modulator M binding in pocket P. The residues in the pocket have restricted motion and are less able to vibrate around their equilibrium positions. Our method sought to approximate the effect of ligand binding by artificially restricting the flexibility of residues in a pocket using a higher spring constant in the elastic network

The reduction in flexibility of an allosteric pocket on modulator binding is shown in Fig. 1. To model this, the unperturbed normal modes were first calculated for the protein. The calculation was then repeated, each time perturbing one of the pockets in the protein. If either atom *i* or *j* was in the pocket to be perturbed then a higher value of 1.5 kcal mol^-1^ Å^-2^ for *k* (1.5 times the previous value) was used instead. This higher value was chosen after values from 1.2–2.5 kcal mol^-1^ Å^-2^ were examined. Active site residues were not counted as being in any pocket for this alteration of *k*, in order to avoid direct perturbation of the site at which the effect was measured. This approach assumes nothing about the shape of the modulator other than that it affects the flexibility of the whole pocket to which it binds.

Once the perturbed NMA had been carried out, the degree of change caused by the perturbation needed to be measured. Since changes at the active site will likely determine how strong an effect a modulator has, the effect of the perturbation on the active site should be considered. Within each individual normal mode the effect of the perturbation was measured by averaging across all identified active site residues the magnitude of the difference between the perturbed and the unperturbed displacements from equilibrium. This is given by:

$$v_{i} = \frac{1}{N_{a}} \sum_{j=1}^{N_{a}} \left | \mathbf{p_{j}} - \mathbf{u_{j}} \right | $$

where *v*_*i*_ is the effect of the perturbation in normal mode *i*, *p*_*j*_ is the displacement of residue *j* in the perturbed normal mode, *u*_*j*_ is the displacement of residue *j* in the unperturbed normal mode, and *N*_*a*_ is the number of active site residues.

The effects of the perturbation within each normal mode then needed to be averaged across the modes in order to get a single numeric measure for the strength of the effect arising from perturbation at one pocket. The effect within each of the normal modes was weighted by the frequency such that the lowest-frequency mode of the chosen modes had the greatest influence on the results. The equation to determine the effect of a perturbation *C*_*m*_ is:

$$C_{m} = \sum_{i=1}^{m} \frac{v_{i}}{\omega_{i}} $$

where *v*_*i*_ is defined above, *ω*_*i*_ is the frequency of mode *i* and is hence equal to the square root of the eigenvalue *E*_*i*_, and *m* is the number of normal modes chosen for the calculation. The justification for this method was that lower-frequency modes within the range selected are likely to be more important in allosteric communication because they consist of the long-range motions of many atoms [21].

It might be expected that larger pockets will have a higher *C*_*m*_ value simply by virtue of having more residues perturbed. In order to account for this a second measure, *E*_*m*_, was defined as:

$$E_{m} = \frac{C_{m}}{N_{p}} $$

where *N*_*p*_ is the number of residues in the pocket and *C*_*m*_ was defined previously. *E*_*m*_ is a measure of the amount of change caused at the active site per residue in the perturbed pocket. A Python script utilising the ProDy package [31] was used to perform NMA on the proteins.

### Machine learning

Values of *C*_*m*_ and *E*_*m*_ with *m* equal to 20, 50, 100, 200 and all modes were chosen as features in a SVM. The features from the Fpocket output used in the SVM were:

- Rank
- Score
- Druggability score
- Number of alpha spheres
- Total SASA
- Polar SASA
- Apolar SASA
- Volume
- Mean local hydrophobic density
- Mean alpha sphere radius
- Mean alpha sphere solvent accessibility
- Apolar alpha sphere proportion
- Hydrophobicity score
- Volume score
- Polarity score
- Charge score
- Proportion of polar atoms
- Alpha sphere density
- Centre of mass - alpha sphere max distance
- Flexibility

See the Fpocket documentation for more details on each of these measures. Distance to the active site, number of residues in the pocket and number of pockets in the protein were also used as features. The distance to the active site for each pocket was calculated as the distance between the geometric centre of the active site residues and the geometric centre of the residues in the pocket. Each feature (apart from number of pockets) was utilised in two different ways: the feature value normalised across all proteins (*raw*); and the ranking of the feature value within the values for that protein, where the ranks were scaled between 0 and 1 (*ranked*).

The 65 features were ranked in Weka explorer [32] using the ChiSquared attribute evaluator and the Ranker search method. This evaluates the worth of a feature by computing the value of the chi-squared statistic with respect to the class. The top 7 features only were retained, as features below this added little value. The retained features, in descending order of descriptive power, were:

- Number of alpha spheres (raw)
- *E*_200_ (ranked)
- Score (raw)
- *E*_*all*_ (ranked)
- Distance to active site (raw)
- Pocket size (raw)
- Fpocket rank (raw)

The SVM-Light package [33] was used to run the SVM. The Gaussian kernel was selected, containing internal parameters *C* and *γ*. The cost factor by which training errors on positive examples outweigh errors on negative examples was set as the ratio of negative to positive examples in the training set (6.19). A leave-one-out parameterisation procedure was carried out over a grid of parameters with *C* equal to 0.01, 0.1, 1 or 10 and *γ* equal to 10^−3^, 10^−4^ or 10^−5^. The procedure consisted of training the SVM on pockets from 78 of the 79 proteins in the training set and testing on pockets from the one left out. The process was repeated for each protein in the set. Performance was similar across the parameter range, with the parameters *C*=1 and *γ*=10^−4^ being selected for the final SVM. Due to the low number of allosteric pockets on each protein, only the top prediction was chosen as being allosteric.

### Web server

**Fig. 2:**
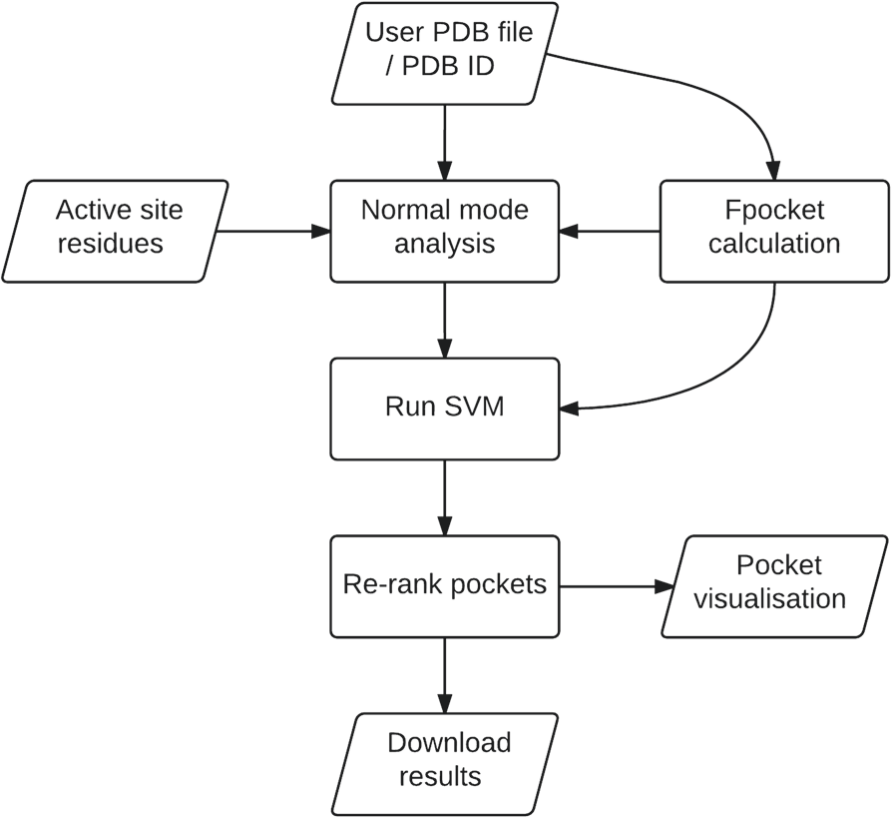
AlloPred pipeline. Flowchart showing the stages involved in running a job submitted to the AlloPred web server. Trapeziums represent inputs or outputs available to the user via the web front end. Rectangles represent stages in the calculation pipeline that occur via the web back end

A flowchart outlining the process of running a job is shown in Fig. 2. The web server was implemented using the Django extension to Python and a SQLite database. JSmol, a JavaScript implementation of the Jmol package, was used for molecular visualisation. Bootstrap was used for page styling. The standalone version of the code runs faster and it is recommended that users who intend to use the method extensively or in batch download the code for local use.

## Results

**Fig. 3:**
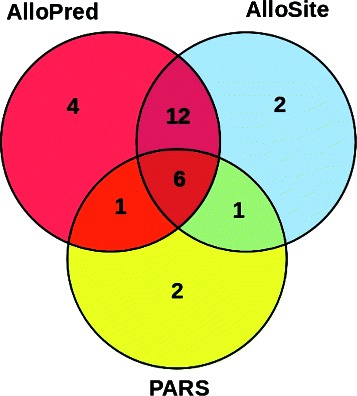
Results comparison by method. Venn diagram showing the number of top predictions for each protein by each method that were correct, from the test set of 40 proteins. For AlloPred and AlloSite a correct prediction was prediction of a pocket containing at least one allosteric binding residue. For PARS a correct prediction was prediction of a site within 10 Å of at least one atom of the allosteric modulator in the protein-modulator crystal

AlloPred was tested on a test set of 40 known allosteric proteins (see the Methods section for selection criteria). For each protein AlloPred ranked the pockets and the top ranked pocket was examined. For 23 of 40 proteins AlloPred ranked top a pocket containing an allosteric binding residue (an *allosteric pocket*), when 18 % of pockets were allosteric pockets. For 28 of 40 proteins an allosteric pocket was ranked first or second. The results were compared to two existing methods for allosteric site prediction. The AlloSite server uses the Fpocket algorithm and a machine learning approach [11], whereas the PARS server combines changes in protein flexibility and a structural conservation score [18]. The correct predictions made by each method, and the overlap between the methods, are shown in Fig. 3. AlloSite ranked an allosteric pocket top in 21 of 40 cases and is suitable for direct comparison to AlloPred as both methods rank pockets from Fpocket. PARS, however, makes predictions of single points; a point was considered allosteric for our purposes if it was within 10 Å of an allosteric modulator atom in the protein-modulator crystal structure. It is important to note the different criteria for a correct prediction when considering the results. PARS ranked an allosteric pocket top in 10 of 40 cases. Figure 3 shows that AlloPred compares well to other methods and makes 4 correct predictions that neither of the other methods do. This suggests that users of other allosteric prediction methods would benefit from the additional use of AlloPred.

In order to reduce the effects of bias during the split of the dataset into training and test sets, the dataset of 119 proteins was additionally split randomly 20 times into training and tests sets of 79 and 40 proteins respectively. The SVM was then trained on the training set, using the previous parameters, and tested on the test set. The average number of correct predictions across the 20 runs was 23.6 out of 40. This shows that the above results used for comparison to other methods are indicative of the performance of the method.

### Web server

**Fig. 4:**
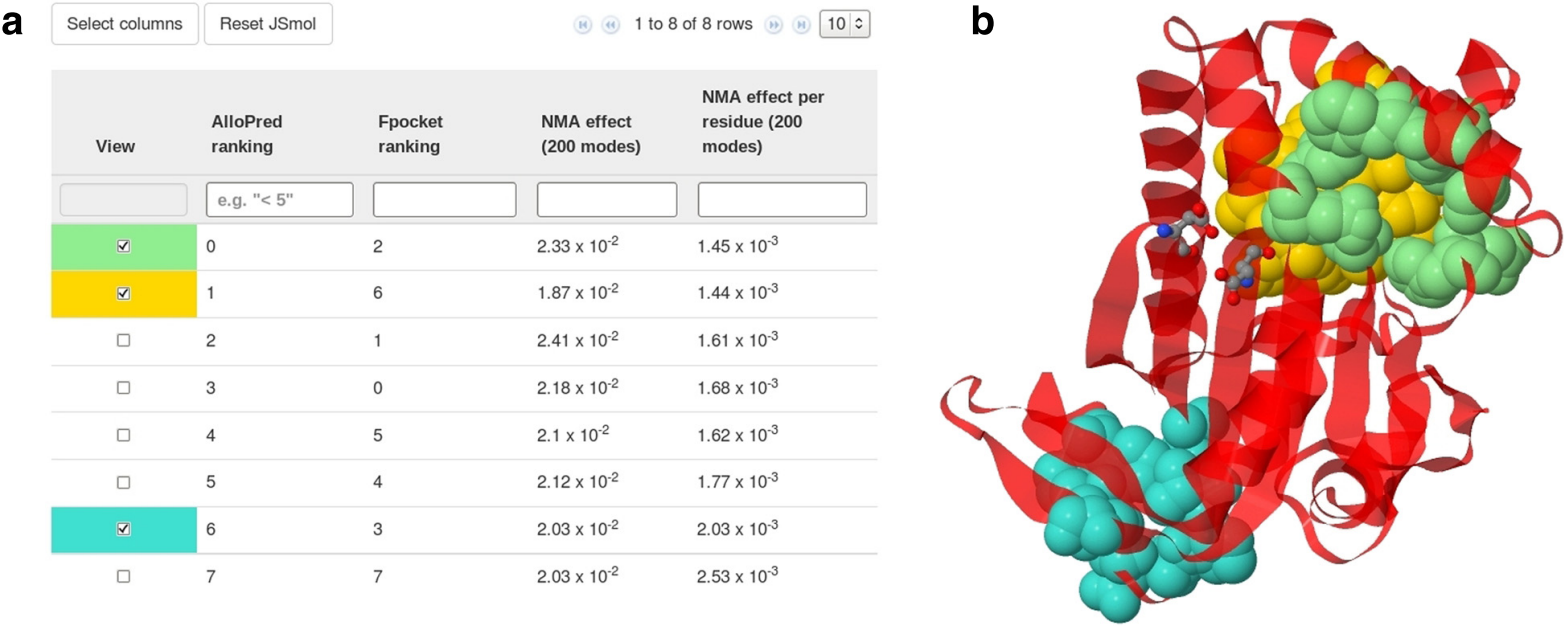
AlloPred results page. Screenshots of the AlloPred results page for the receptor-type adenylate cyclase with PDB ID 1FX2. **a** The results table with default columns selected. Three pockets have been chosen for visualisation. **b** The JSmol window shown when the boxes are selected as in (**a**). The ribbon visualisation option is used and the residues identified as being part of the active site are shown as balls and sticks. Three pockets are shown in green, yellow and blue. AlloPred correctly predicts the green pocket as being allosteric

The AlloPred web server allows users to analyse the prediction results via an intuitive interface. Users can either input a PDB ID and chain(s) or upload a PDB file. The active site residues of the protein must be given but there is an option to retrieve this data, if it is available, from the Catalytic Site Atlas [29]. The results page is shown in Fig. 4. All pockets are displayed in a table with their AlloPred rankings and Fpocket output. The table can be sorted and filtered by any one or more of the 29 AlloPred and Fpocket features. The page also allows users to visualise each pocket on the protein in a JSmol window that lets the user explore the protein and its predicted allosteric sites. Features include highlighting the active site residues, selecting one of three visualisation options and a JSmol terminal to insert custom commands. The results, including full details of each pocket, can be downloaded for further analysis as a tab-delimited text file. The calculation time is fast, with a 400 residue protein (∼15 predicted pockets) analysed within 5 min.

## Discussion

Over the last few years a renewed interest in allostery, perhaps due to the potential benefits of allosteric drugs, has led to the development of a number of computational approaches to understanding allostery [25]. Some of these are directly associated with predicting allosteric sites on proteins from structure alone.

The AlloSite server is similar to the method presented here in that it uses the Fpocket algorithm and attempts to elucidate allosteric pockets [11]. Whereas AlloSite solely uses the Fpocket output, our method uses an approach that combines flexibility with the Fpocket output. A combination of methods may give better predictions than either method individually, as indicated by the unique predictions made by both methods during testing. In fact the AlloSite predictions were found in every case to correspond to the pocket ranked top by Fpocket. The complete ranking of pockets provided by AlloPred may also be useful, as pockets ranked second were often found to be allosteric in the test set.

An approach that combines flexibility analysis using normal modes and structural conservation scores [10] is also similar to the method presented here and was recently turned into a web server, PARS [18]. Although direct comparison is difficult due to the differences in site calculation, definition of allosteric sites and datasets used, the method presented here again may be used well in combination as shown by Fig. 3.

The lack of input about the shape of the ligand and the large coverage of the protein in terms of pockets (average 18.5 pockets per protein) used by our method mean that it may be able to predict novel or unusual sites that methods which explicitly model the modulator might not. This is important, for example when searching for allosteric sites on proteins believed to be non-allosteric. The lack of conservation-based approaches in our method also facilitates discovery of sites not currently preserved by evolution. This is useful due to the large variety of allosteric modulators [34] and mechanisms [3], suggesting potential novel modulators for proteins with known allosteric pathways.

Other promising approaches [15, 17, 19] investigate the allosteric pathway and are not directly comparable with this method, which is only concerned with how the pathways transmit the effects of perturbations to the normal modes and does not directly reveal any information about the pathways themselves. Again, a combination of our method with these approaches may be useful, as pockets predicted using our or other methods can be further investigated to reveal information about the underlying allosteric communication.

The main limitation of our method is related to the diversity found in allosteric systems. Rigid-body motions of oligomers, side-chain dynamics, backbone motions and local unfolding are all mechanisms of allostery, with allosteric effects even present in intrinsically-disordered proteins [3]. A method based around the changes in dynamics on ligand binding is likely to miss many allosteric effects, and this can go some way to explaining the predictions of our method that were incorrect. In particular, classic examples of allostery such as haemoglobin that involve oligomeric re-organisation to affect ligand cooperativity are not suitable for use with this method. However, the results shown here and in other studies are encouraging and indicate a future where we can pick modulating sites on proteins with reasonable confidence. Our method, for example, successfully predicts allosteric sites on proteins with a variety of sizes and functions.

## Conclusions

A machine learning approach that utilises normal mode analysis and pocket descriptors to predict allosteric pockets on proteins was developed and tested on a set of known allosteric proteins. The method was able to pick out pockets containing one or more allosteric residues. The new approach presented here is comparable in performance to existing methods and has the potential to find novel allosteric sites due to its high coverage of the protein surface and lack of information about the ligand shape. It also exhibits complementarity with existing methods. The web server provides features for visualisation and analysis that allow exploration of the results in a manner that other servers do not.

The generalisation of allosteric site prediction methods from individual proteins to the whole of protein space has only begun in earnest in recent years but is the first step on the path to effective virtual screening for allosteric drugs. Without such site prediction methods, the vast potential of allosteric drugs as therapeutics will remain untapped.
